# Real-Time Measurement of Width and Height of Weld Beads in GMAW Processes

**DOI:** 10.3390/s16091500

**Published:** 2016-09-15

**Authors:** Jesús Emilio Pinto-Lopera, José Mauricio S. T. Motta, Sadek Crisostomo Absi Alfaro

**Affiliations:** 1 Universidad Católica de Manizales, Facultad de Ingeniería y Arquitectura, Unidad Académica de Ciencias Naturales y Matemáticas, Manizales 170001, Colombia; jpinto@ucm.edu.co; 2 Automation and Control Group in Manufacturing Processes—GRACO, Department of Mechanical Engineering, University of Brasilia, Brasilia DF 7191090, Brazil; jmmotta@unb.br

**Keywords:** monitoring of GMAW process, weld bead geometry, passive vision, digital images processing, real-Time

## Abstract

Associated to the weld quality, the weld bead geometry is one of the most important parameters in welding processes. It is a significant requirement in a welding project, especially in automatic welding systems where a specific width, height, or penetration of weld bead is needed. This paper presents a novel technique for real-time measuring of the width and height of weld beads in gas metal arc welding (GMAW) using a single high-speed camera and a long-pass optical filter in a passive vision system. The measuring method is based on digital image processing techniques and the image calibration process is based on projective transformations. The measurement process takes less than 3 milliseconds per image, which allows a transfer rate of more than 300 frames per second. The proposed methodology can be used in any metal transfer mode of a gas metal arc welding process and does not have occlusion problems. The responses of the measurement system, presented here, are in a good agreement with off-line data collected by a common laser-based 3D scanner. Each measurement is compare using a statistical Welch’s *t*-test of the null hypothesis, which, in any case, does not exceed the threshold of significance level α = 0.01, validating the results and the performance of the proposed vision system.

## 1. Introduction

The gas metal arc welding (GMAW) process is widely used in industry due to its ease of automation and high productivity. This process uses a continuously-fed consumable metal wire electrode, and all commercially important metals can be welded in all positions by choosing appropriate welding process variables, such as wire feed rate and voltage, and consumables, such as shielding gas and electrodes. In general, the process variables must be set properly to attain a weld bead with the required appearance and quality. A good approach to achieve better welding parameters, and thus better weld beads, is relating the process response directly to different input variables through a control system. In the case of feedback control of the weld bead geometry, the monitoring of variables of interest—for example, width and height—is necessary.

A common method to monitor geometric variables in GMAW processes is the indirect mode, by using a mathematical model to relate the geometric parameters to another process variable that is easier to measure, such as current or voltage [1,2]. For example, penetration is an internal parameter and, in general, it is estimated by indirect methods [3,4]. Despite the fact that direct measurement approaches such as ultrasound [5] and computerized radiographic [6] exhibit good results, these methods are difficult to perform in a welding process. Indirect measurement approaches are useful when direct measurement is not possible, but they increase the error in the monitoring process and can only be used in the same conditions as those in which the modeling development was conceived. On the other hand, computer vision systems are used to directly measure, online, the external geometric parameters of the weld bead. The main problem with these systems is the unfavorable visualization of the weld pool due to the strong radiation generated from the electric arc in the welding process. For this reason, and in order to reduce the problem, two approaches are employed: systems based on structured light and systems based on optical filters. In each case, two different techniques are used to measure variables of interest. In structured light systems, a single laser-stripe can be projected directly onto the weld bead [7,8,9,10,11]; these systems are easily implemented, they can reconstruct the weld bead surface, and show good results, but they have a significant spatial delay with respect to the weld bead formation, causing complications in real-time control systems. Alternatively, structured laser-light pattern systems, such as parallel stripes [12], grids [13], and dot-matrix projections [14] are used. These systems project the laser patterns directly onto the weld pool and employ the specular reflection of the laser-light to describe the weld bead formation. Structured light patterns provide meaningful information about the geometric parameters, but in practice they are restricted to the gas tungsten arc welding (GTAW) process, because the reflected pattern is very affected by fluctuations of the weld pool surface, which are common in welding processes with metal transfer such as in GMAW.

In the case of systems based on optical filters, active or passive vision systems can be found. Active systems employ an external illumination source and optical band-pass filters, with a narrow bandwidth according to the light-source [15,16]. In general, infrared laser is used as an illumination source based on its lower noise level with respect to the visible spectrum and the electric arc. These systems acquire weld pool images with high quality, especially in GTAW processes, but they are not commonly used in control processes because of their additional costs and complexity. On the other hand, passive systems do not use external illumination. The use of optical filters and the existence of radiation from the arc and the weld pool are sufficient to extract geometrical features from the images. Although the captured images from the weld pool with active vision systems are more detailed, in passive vision the high contrast between the weld pool and the other objects in the scene is in favor of suitable results from measurements and seam tracking processes [17,18].

Concerning the methodologies for measuring external geometric parameters in GMAW processes, such as width and height of the weld beads, top-view images from the weld bead are commonly captured for measuring width [17,18]. When measuring the weld bead height, cameras with a side-view of the workpiece and optical axis parallel to the workpiece surface are used. Two cameras are thus needed to monitor both width and height parameters [19]. The employment of a single camera for measuring the width and height at the same time is presented by Zhang et al. [20], who achieved a 3D model reconstruction of the weld pool in pulsed GMAW. They used the camera sensor parallel to the welding direction, therefore the width and height measurements depended on the angle between the camera optical axis and the workpiece plane. The disadvantages of this method were the need for precise alignment and the susceptibility to occlusions of the weld pool by the gas nozzle of the welding torch in natural metal transfer modes. Among the main digital image processing tools used to find regions and feature points, smoothing filters are applied to enhance the image. Analysis of the gray scale gradient [20], Laplacian operator [18], or Sobel operator [19] are commonly employed in the edge detection process. Thresholding methods [17] or the Hough Transform are used to detect the borders of the weld bead [18,19] or weld pool [20].

This paper presents a passive vision system for directly measuring the width and height of weld beads in real-time in a GMAW process. In this work, a single camera is used to measure both geometric parameters. The vision camera is directed and focused to the weld pool, the vision system uses the point with the lowest temperature at the rear of the weld pool for measuring height and finds the weld pool edge for measuring width. Reference points in the image are found using local thresholding and connected components labeling. The calibration process is achieved by using projective transformations and homography matrices. The camera location and the proposed system, focused in the weld pool, allow capturing of the image without occlusion problems and allow measuring the width and height with a processing time less than 3 milliseconds per image, which permits the use of the vision system in futures real-time adaptive control systems of the geometric parameters of weld beads in GMAW processes. The data collected in different experiments are in a good agreement with an off-line laser-based 3D scanner (single laser-stripe) employed to validate the system.

## 2. Materials and Methods

### 2.1. Measurement Process

**Figure 1:**
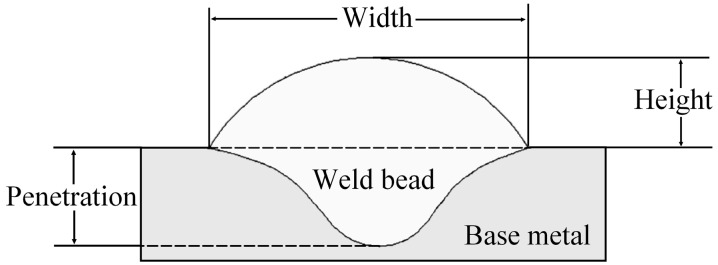
Weld bead geometric characteristics (cross-section).

Figure 1 illustrates the cross-section of a weld bead with its main geometric features. The width and height are the external variables and the target geometrical feature of this work.

The measurement methodology involves three stages in the system setup:

- Calibration process;
- Image processing;
- Image acquisition.

#### 2.1.1. Image Acquisition

**Figure 2:**
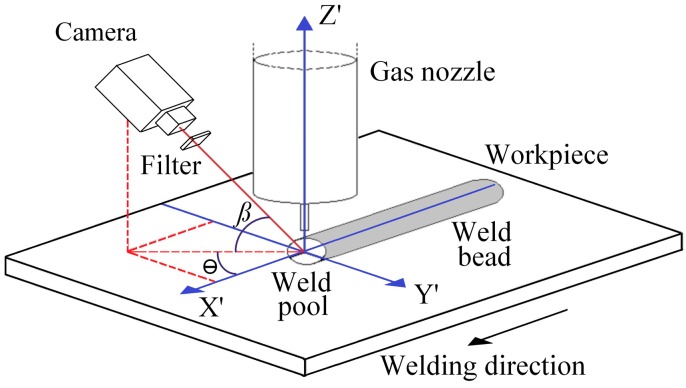
Image acquisition setup including the world coordinate system.

Figure 2 shows an overview of the image acquisition setup with the world coordinate system represented by the perpendicular axes X’, Y’, and Z’. The welding direction is along the X’ axis, on the symmetry axis of the weld bead, the Y’ axis is on the surface of the workpiece plane and Z’ axis is along the electrode-wire axis.

The camera is fixed in front of the weld pool with an angle *β* between the optical axis of the camera and the X’-Y’ coordinate plane axes, and an angle *θ* between the X’ axis and the projection of the same optical axis on the workpiece plane. In this case, the angle *θ* allows an appropriate decrease of the angle *β*, obtaining a view of the weld pool without losing information, whatever the distance between the contact tip and the workpiece is. Angles *β* and *θ* can take values between 20° and 70°. In this work the angles used were approximately 30° for *β* and 50° for *θ*.

**Figure 3:**
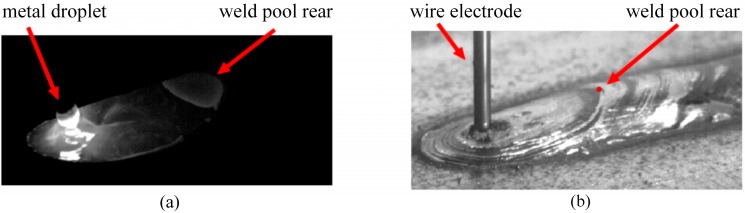
Reference points and objects in the image: (**a**) Typical frame acquired with the actual image acquisition system; (**b**) Off-line frame illustrating the weld pool rear and the corresponding reference point in the weld bead.

To eliminate the more intense radiations from the electric arc and the weld pool in the visible light range of the electromagnetic spectrum, the vision system uses a long-pass optical filter with a threshold at 800 nm. The images are captured with a CMOS (Complementary Metal-Oxide Semiconducor) high-speed camera with an exposure time of 50 μs and resolution of 416 × 200 pixels with 256 gray levels and a frame rate of 100 frames per second (fps). Figure 3a shows a typical frame acquired with this system, in a short-circuiting transfer mode. As reference, Figure 3b presents an off-line frame to illustrate the weld pool rear and the weld bead.

#### 2.1.2. Calibration Process

**Figure 4:**
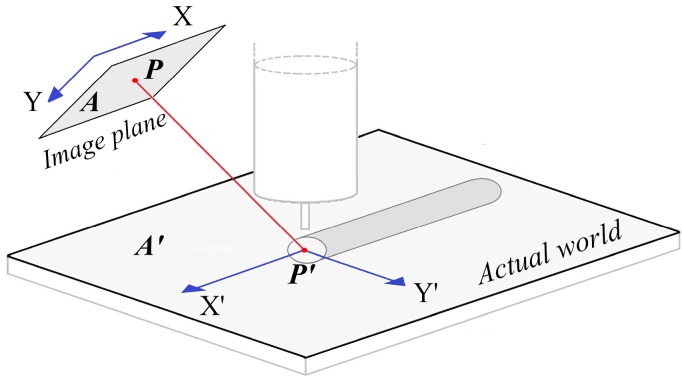
Corresponding points between the world and image plane coordinates.

In this work, and according to Figure 2, the X’-Y’ plane was used to measure the width and the X’-Z’ plane was used to measure the height, then, two 2D projective transformations wee used to calibrate each plane. A 2D projective transformation can establish a relationship between the corresponding points in the image of a flat object and the actual world through a mapping performed by a homography matrix. As described in Hartley and Zisserman [21] and depicted Figure 4, a planar projective transformation is a linear transformation on homogeneous 3-vectors represented by a non-singular 3 × 3 matrix:

(1) $$\left(\begin{array}{c} x_{i}^{\prime }\\ y_{i}^{\prime }\\ w_{i}^{\prime } \end{array}\right)\;=\left[\begin{array}{ccc} h_{11} & h_{12} & h_{13}\\ h_{21} & h_{22} & h_{23}\\ h_{31} & h_{32} & h_{33} \end{array}\right]\left(\begin{array}{c} x_{i}\\ y_{i}\\ w_{i} \end{array}\right)$$

which can be expressed more briefly as ***P’*** = ***H.P***, where ***H*** is the homography matrix. In this case, any 2D point ***P’_i_*** = ($X_{i}^{\prime }$, $Y_{i}^{\prime }$) in the plane ***A’*** of the actual world (Figure 4) can be represented as a homogeneous 3-vector ${P^{\prime }}_{i}={\left(x_{i}^{\prime },y_{i}^{\prime },w_{i}^{\prime }\right)}^{T}$, and the corresponding point ***P_i_*** in the image plane ***A*** can be represented by the homogeneous 3-vector $P_{i}={\left(x_{i},y_{i},w_{i}\right)}^{T}$.

Here, in the image plane $w_{i}=1$, and the nonhomogeneous coordinates of any point in the actual world are expressed as:

(2) $$X_{i}^{\prime }=\;x_{i}^{\prime }/w_{i}^{\prime }\;Y_{i}^{\prime }=\;y_{i}^{\prime }/w_{i}^{\prime }$$

A homography matrix can be estimated using four point correspondences and solving the matrix system:

(3) Mh=0

where h is a column vector with the nine entries of the homography matrix ***H***:

(4) $$h={\left(h_{11}\;h_{12}\;h_{13}\;h_{21}\;h_{22}\;h_{23\;}h_{31}\;h_{32}\;h_{33}\right)}^{T}$$

and

(5) $$M=[\begin{array}{ccccccccc} x_{1} & y_{1} & 1 & 0 & 0 & 0 & -x_{1}x_{1}^{\prime } & -y_{1}x_{1}^{\prime } & -x_{1}^{\prime }\\ 0 & 0 & 0 & x_{1} & y_{1} & 1 & -x_{1}y_{1}^{\prime } & -y_{1}y_{1}^{\prime } & -y_{1}^{\prime }\\ x_{2} & y_{2} & 1 & 0 & 0 & 0 & -x_{2}x_{2}^{\prime } & -y_{2}x_{2}^{\prime } & -x_{2}^{\prime }\\ 0 & 0 & 0 & x_{2} & y_{2} & 1 & -x_{2}y_{2}^{\prime } & -y_{2}y_{2}^{\prime } & -y_{2}^{\prime }\\ x_{3} & y_{3} & 1 & 0 & 0 & 0 & -x_{3}x_{3}^{\prime } & -y_{3}x_{3}^{\prime } & -x_{3}^{\prime }\\ 0 & 0 & 0 & x_{3} & y_{3} & 1 & -x_{3}y_{3}^{\prime } & -y_{3}y_{3}^{\prime } & -y_{3}^{\prime }\\ x_{4} & y_{4} & 1 & 0 & 0 & 0 & -x_{4}x_{4}^{\prime } & -y_{4}x_{4}^{\prime } & -x_{4}^{\prime }\\ 0 & 0 & 0 & x_{4} & y_{4} & 1 & -x_{4}y_{4}^{\prime } & -y_{4}y_{4}^{\prime } & -y_{4}^{\prime } \end{array}]$$

The matrix system of Equation (3) can be solved using the *Singular Value Decomposition* (SVD) of ***M***, i.e., if $M=UDV^{T}$, then the last column of V is the vector h. In the same way, the estimation process can be used with more than four point correspondences to ensure a more robust solution (overdetermined system), with the restriction that no more than two points are collinear.

**Figure 5:**
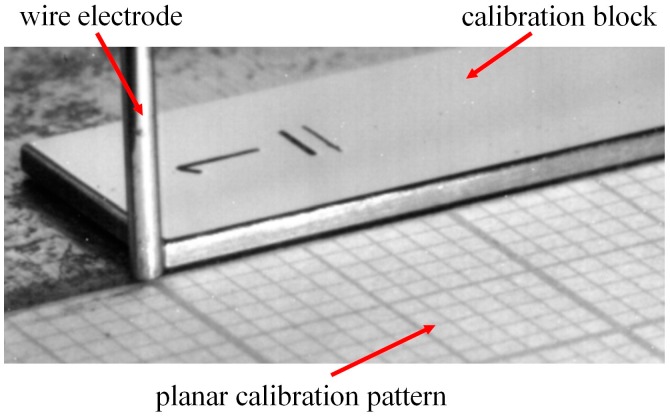
Illustration of planar pattern and calibration block used [22].

In this work the reference point X’ = Y’ = Z’ = 0 was fixed at the intersection of the three perpendicular axes X’, Y’, and Z’ (Figure 2). The image calibration process was performed by using a planar calibration pattern for the X’-Y’ plane and different calibration blocks for the X’-Z’ plane, as shown in Figure 5. According to Figure 5, each square in the planar pattern corresponds to 1 mm^2^, the height of the calibration block is aproximatelly 1 mm, and the spatial resolution along the X’ axis is approximatelly 0.08 mm/pixel in the X’-Y’ plane and 0.05 mm/pixel in the X’-Z’ plane. The wire electrode is used as a reference in the image.

Six reference points were used to calibrate the image in each of the planes. The X’-Y’ plane was calibrated from 0 to 2 mm along the weld bead direction (X’ axis) and from 0 to 6 mm in the Y’ axis. The X’-Z’ plane was calibrated in the Z’ direction by using two blocks, 1.5 mm and 3 mm, from 0 to 15 mm in the weld bead direction (X’ axis). Reference points in the X’-Y’ plane and X’-Z’ plane were used independently to generate the corresponding matrix ***M*** in the form of Equation (5). Then, the SVD was used in each case to solve the matrix system of Equation (3) to find the respective homography matrix. Point positions in the image plane were translated to actual real world positions in each of the planes, X’-Y’ and X’-Z’, by using Equation (1), and to nonhomogeneous coordinates by using Equation (2).

#### 2.1.3. Image Processing Methodology

In general, measurements of width and height are performed by using a *thresholding* technique. For each case, before the thresholding step, an image *enhancement* is employed to ensure a good segmentation of the weld pool, and at last, a *labeling* technique is used to identify reference points in the weld pool and to acquire a real-time data set. Aiming to reduce the computer processing time to measure both parameters, a vertical axis in the image plane was used to split each of the frames into two regions: the left one was used to measure the width and the right one to measure the height. Although the two measured parameters (width and height) were obtained from different approaches, the image processing methodology in both cases comprises the following image processing routines: image enhancement; image thresholding; labeling; and reference point location.

Image enhancement is used mainly to remove noise and it is carried out using a *Gaussian smoothing* filter before measuring the width and a *mean* filter before measuring the height. In the case of height measurement, where the target region is at the rear of the weld pool, a *mean* filter was employed to connect sub-regions separated by chains of pixels with low gray-level values into the weld pool. *Gaussian* and *mean* filters are spatial filters and use a 2D kernel with a convolution operation, so they are linear filters. This convolution operation can be calculated from:

(6) $$I_{A}\left(x,y\right)=\;\overset{\frac{n}{2}}{\underset{h=-\frac{n}{2}}{\sum }}\;\;\;\overset{\frac{n}{2}}{\underset{k=-\frac{n}{2}}{\sum }}A\left(h,k\right)I\left(x-h,y-k\right)$$

where: $I_{A}\left(x,y\right)$ is the new pixel value at the (x,y) coordinates; h and k are the coordinates of each pixel in the mask (kernel); I represents the original image; A is the square mask; n is an odd number of rows or columns of the mask, so the expression n/2 is taken as the smallest integer of division. In this work, the *mean* filter was modeled with a mask of 7 × 7 pixels, and the *Gaussian* filter with a mask of 5 × 5 pixels.

A thresholding technique was employed in image segmentation to remove the background and separate the target objects. A threshold, T∈[0,256], is applied to the input set of pixels in the image and a new set of pixels with only two intensity values was obtained, creating a binary image. In this work, a 0 value was assigned to the background according to the equation:

(7) $$g\left(x,y\right)=\{\begin{array}{l} 0,\;\;\;\mathrm{if}\;\;\;\;f\left(x,y\right)<T\;\\ 1,\;\;\;\mathrm{if}\;\;\;f\left(x,y\right)\geq T \end{array}$$

where g(x,y) is the new value of the output pixel in the image, at row x and column y, and f(x,y) is the input pixel intensity value in the same coordinates before thresholding. In the case of height measurements, the weld bead and the workpiece usually have large differences in the pixel intensity values (gray-levels near 0 on the workpiece region), then a fixed threshold, $T_{H}=10$, wa used.

For width measurements, it is considered that some arc radiation can be reflected in the workpiece around the weld pool where the electric arc has direct contact, then a threshold, $T_{W}$, was estimated as a piecewise function using the following expression:

(8) $$T_{W_{i}}=\{\begin{array}{l} \left(-10*W_{i-1}\right)+100,\;\;\;\mathrm{if}\;\;W_{i-1}<10\;\mathrm{mm}\;\\ 0,\;\;\;\;\;\;\;\;\;\;\;\;\;\;\;\;\;\;\;\;\;\;\;\;\;\;\;\;\;\;\;\;\;\;\;\mathrm{if}\;\;W_{i-1}\;\geq 10\;\mathrm{mm} \end{array}$$

which is a linear equation of the width calculated before ($W_{i-1}$), taking 100 as the highest threshold value. The formulation expresses that for weld beads with a width above 10 mm there is no reflected radiation from the workpiece. It also considers that more radiation will be reflected from the workpiece if the weld bead has small dimensions, therefore a higher threshold value for a good segmentation of the weld pool is needed. Besides, since at the start of the welding process there is no accurate information available about the bead width, a fixed threshold ($T_{W}=0$) was used during the first second of the welding.

In the special case of a short-circuit metal transfer mode, in each frame a threshold was assigned as the mean of the pixel´s intensity values at the arc region (left in the image, Figure 3) aiming at determining whether the processes are operating in an open-arc period or in a short-circuit period. In the case of short-circuit period, both thresholds $T_{H}$ and $T_{W}$ were assigned to be 0. For the open-arc period both thresholds were calculated in the standard mode. As a rule of thumb, automated methods for finding thresholds that only take into account the pixel intensity information are not recommended, because the electric arc and the weld bead images can be overlapped. So, the images may be mistaken as corresponding to the same object, even if they have large differences in intensity, producing wrong results.

Subsequent to the thresholding process, a labeling routine is started in the binary image in such a way that it searches for reference points in both width and height measurements. For measuring width, the search is carried out by crossing the weld pool region in a fixed trajectory until the reference point is identified. For measuring height, the edge of the region of the weld pool rear is tracked and each point in the edge is evaluated to determine the respective reference point.

For height measurement the right region of the image was used. The process starts in the first column (left) and last row (bottom) where the pixel value is 0. The process traverses pixel by pixel until finding a pixel with gray intensity value 1, which is considered as weld pool edge, then, starting from left to right, a connected-component labeling algorithm based on the 8-connectivity traverses only the object edge until again finding the first column of this region. To determine the reference point location, each pixel in the edge is evaluated to find the position with the longest Euclidean distance from the reference point X’ = Y’ = Z’ = 0. In the process, each point position is normalized with respect to the number of rows and columns of the image so as not to have preferences for the row or column position of each evaluated point.

**Figure 6:**
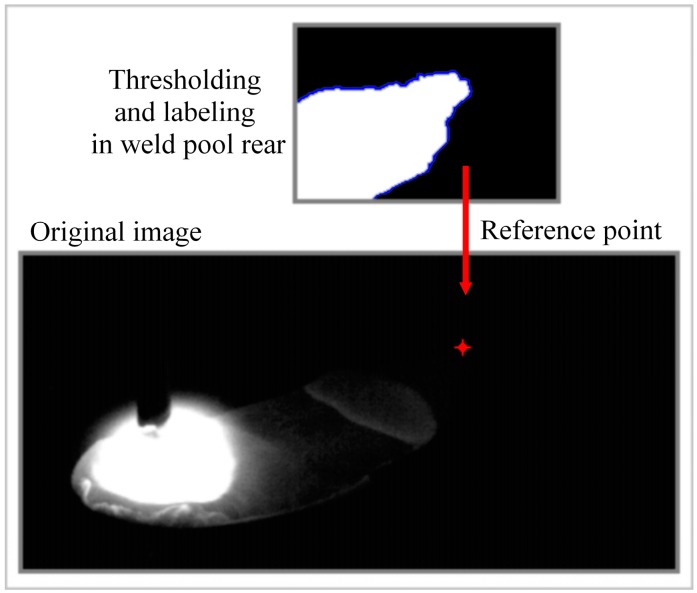
Reference point located for a typical acquired frame and the weld pool rear thresholding and labeling [22].

The reference point determined in the height measurement process is considered as the point with the least temperature at the weld pool rear. This point is accepted as the highest in the weld pool, thus it determines the weld bead height. It is accepted that this reference point is located in the middle of transversal section of the weld bead (in the X’-Z’ plane, Figure 2). As an example, Figure 6 shows a typical acquired frame with a reference point located in the image at row 58 and column 280, in the X’-Z’ plane, with a height of 2.23 mm from the workpiece plane, using a constant voltage of 21 V and a wire feed rate of 6 m/min as welding parameters. Figure 6 also shows the binary image of the weld pool rear after thresholding and indicates the labeling processes across the edge of weld pool in this region.

In order to measure the weld bead width, the process was performed on the weld pool, over the workpiece plane along the Y’ axis (Figure 2). The direction followed during labeling was predefined in the calibration process and follows the Y’ axis direction from the position X’ = Y’ = 0 (in the world coordinates) until the last row in the image. Considering that these positions are in the X’-Y’ plane, the trajectory is fixed, but the process finishes when the edge of the weld pool was found. The labeling process tracks all pixels along the Y’ axis with pixel value g(x,y)=1 (belonging to the weld pool) until a pixel with g(x,y)=0 found. This point is identified as the edge of the weld pool and it is considered as the reference for the measurement process.

**Figure 7:**
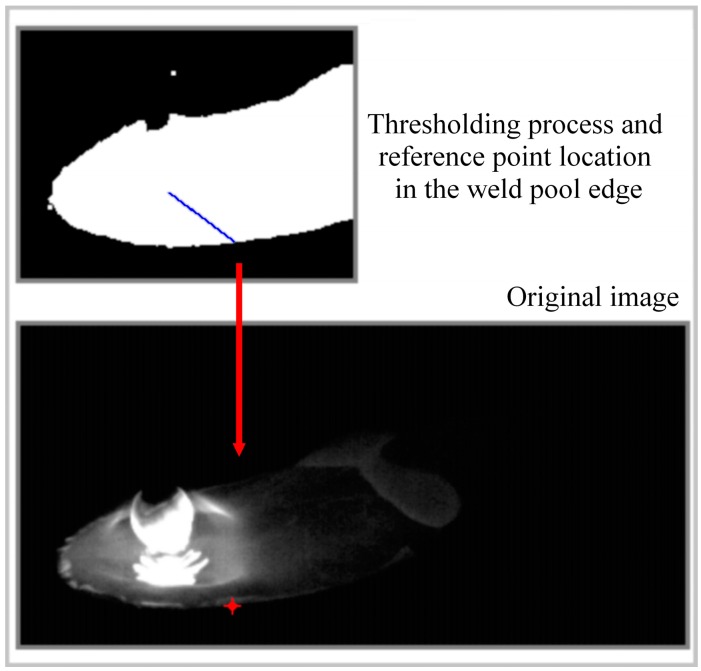
Reference point located in the weld pool edge to measure the weld bead width [22].

In this case, the weld pool was supposed to have a constant width, so computing up to the middle of the bead width was enough to completely measure the width, then the weld bead width was calculated as twice the width value computed at each step. Figure 7 shows an example of the thresholding and indicates the labeling processes in the target region and the reference point located in the original image. In this case, for a welding process with a constant voltage of 23 V and a wire feed rate of 7.5 m/min as input parameters, the reference point position in the image was at row 175 and column 132, resulting in a weld bead width calculated as 7.46 mm.

**Figure 8:**
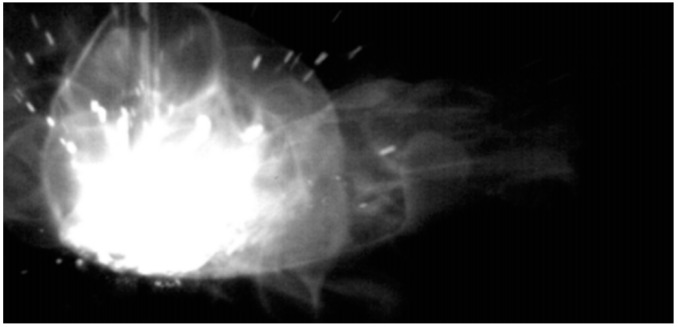
Incidental explosion in short-circuiting metal transfer mode [22].

Finally, data was acquired in real-time as a 2D signal of the measurement results in time. During the process a Gaussian filter was applied in each acquired signal of the collected data set. The filter is employed to minimize possible measurement errors from uncontrollable incidents such as explosions in the short-circuit metal transfer mode, such as in the example showed in Figure 8. In this case, errors in reference point locations are due to an inaccurate thresholding value selection, this due to an uncontrollable and abrupt change in the illumination scene and the nature of the thresholding technique.

### 2.2. Validation Process

The measurement methodology proposed in this article has two fundamental steps: calibration and reference point location. Here, the homography matrix computed in the calibration process depends on the camera position in the image acquisition setup (Figure 2). Aiming at validating the calibration process, different predefined reference points in both X’-Y’ and X’-Z’ planes were used for comparing directly with the developed system results. Concerning reference point location, the validation process was conceived by using different experiments in order to compare the measurement results from the proposed system with a classical off-line laser-based 3D scanner (a single laser-stripe, with resolution of 0.05 mm in each measurement, width and height).

**Figure 9:**
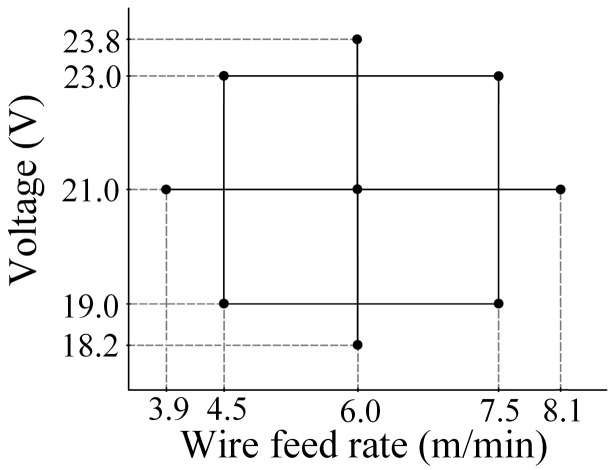
Work points used in validation process.

Figure 9 shows nine experimental work points used in the validation process, each one corresponding to a set of input variables, voltage and wire feed rate. As shown in Figure 9, the work points are distributed using a *central composite design*, with the aim of inspecting the width and height in a stable metal transfer region of the process. The consumable materials employed were: electrode AWS A5.18 ER70S-6 with 1.2 mm diameter; steel plates AISI 1020; shielding gas Ar + 6% CO_2_ with a flow rate of 15 L/min. The power source was used in a constant voltage mode with a distance from contact tip to the workpiece of 15 mm.

Supposing a region of stable metal transfer in the process, the width and height of a weld bead in a current weld shall be uniform. Therefore, a statistical test can be used to compare measurements carried out by the proposed vision system with measurements from the 3D scanner. A common test of the null hypothesis is the Student’s *t*-test when each of the means of two populations are equal. This statistical test can only be used if the variances of the two populations are supposed equal. If this is not the case, a Welch’s *t*-test can be used, since it is indifferent to the equality of the variance. Welch’s *t*-test can be employed for unpaired and paired samples, but with the assumption that each population evaluated should follow a normal distribution (parametric test). In the case of unpaired samples, the statistic t for the Welch’s *t*-test is defined as:

(9) $$t=\frac{\mu_{1}-\mu_{2}}{\sqrt{\frac{s_{1}^{2}}{n_{1}}+\frac{s_{2}^{2}}{n_{2}}}}$$

where *µ*_1_ and *µ*_2_ are the respective sample means, $s_{1}^{2}$ and $s_{2}^{2}$ are the sample variance and $n_{1}$ and $n_{1}$ are the sample sizes. The degrees of freedom associated with the test can be calculated as:

(10) $$\nu =\frac{{\left(\frac{s_{1}^{2}}{n_{1}}+\frac{s_{2}^{2}}{n_{2}}\right)}^{2}}{\frac{{\left(s_{1}^{2}/n_{1}\right)}^{2}}{n_{1}-1}+\frac{{\left(s_{2}^{2}/n_{2}\right)}^{2}}{n_{2}-1}}$$

then, |t| and ν (rounded down to the nearest integer) can be used with the Student’s t-distribution to test if two population means are equal (usually for *p-values* less than 0.10, 0.05, or 0.01). For paired populations, Equations (9) and (10) can be used in the same way, but using the mean of the residuals for the two populations in the numerator.

## 3. Results and Discussions

**Figure 10:**
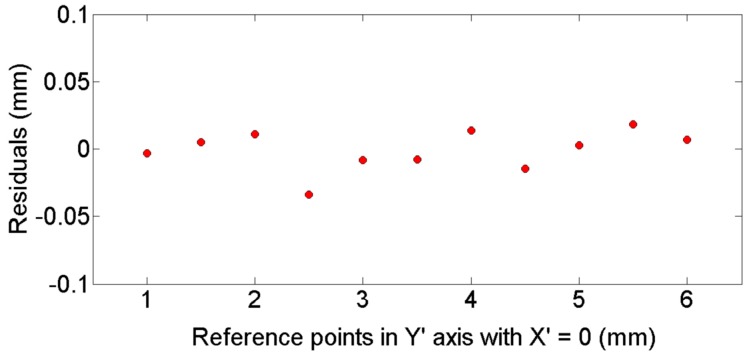
Residual of reference point positions along the Y’ axis with constant X’ = 0 [22].

**Figure 11:**
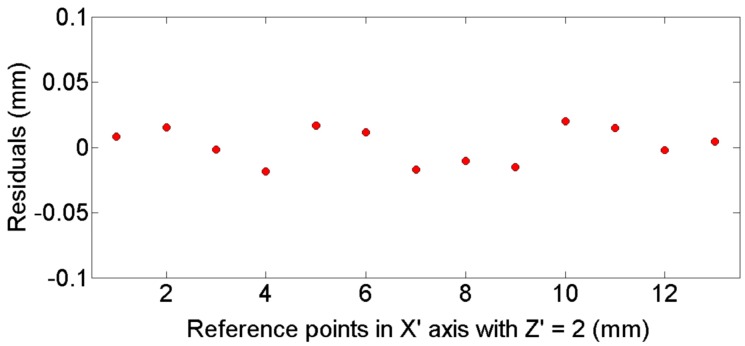
Residual of reference point positions along the X’ axis using the 2 mm calibration block [22].

Figure 10 shows a residuals plot of different positions computed along the Y’ axis for X’ = 0. Figure 11 plots the residuals for a constant height value of Z’ = 2 mm, along different positions in X’ axis. Figure 10 and Figure 11 indicate an adequate distribution of residuals around 0 in both cases, with similar standard deviations *s* = 0.01 mm. Similar results are obtained using different reference points in each plane (X’-Y’ and X’-Z’).

**Figure 12:**
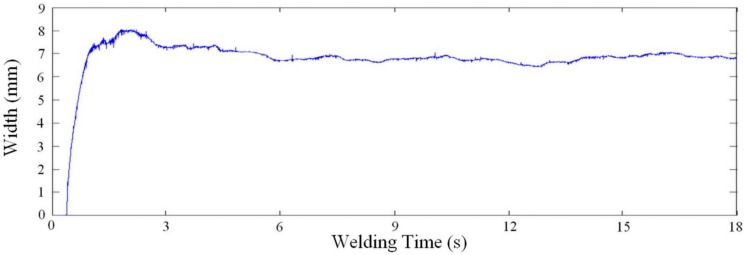
Real-time width measurement for a work point of 21 V and 6 m/min, using a welding speed of 10 mm/s and 100 fps as sampling rate [22].

**Figure 13:**
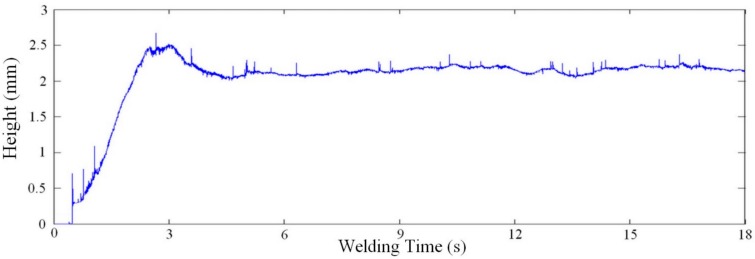
Real-time height measurement for a work point of 21 V and 6 m/min, using a welding speed of 10 mm/s and 100 fps as sampling rate [22].

Figure 12 and Figure 13 show, respectively, a typical response of the proposed system for width and height measurements with a work point of 21 V and 6 m/min, for a welding speed of 10 mm/s. In this case, the measurements of width and height is 6.79 mm (*s* = 0.34 mm) and 2.15 mm (*s* = 0.09 mm), respectively, measured after four seconds (time established as sufficient for the welding process stabilizes after the ignition).

**Figure 14:**
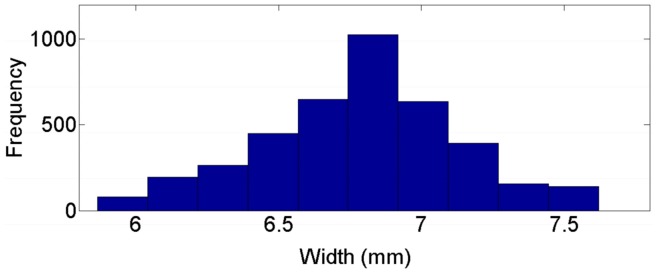
Frequency distribution for width measurements with a work point of 21 V and 6 m/min.

**Figure 15:**
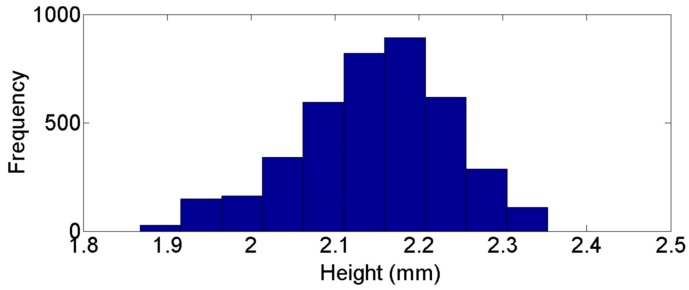
Frequency distribution for height measurements with a work point of 21 V and 6 m/min.

**Figure 16:**
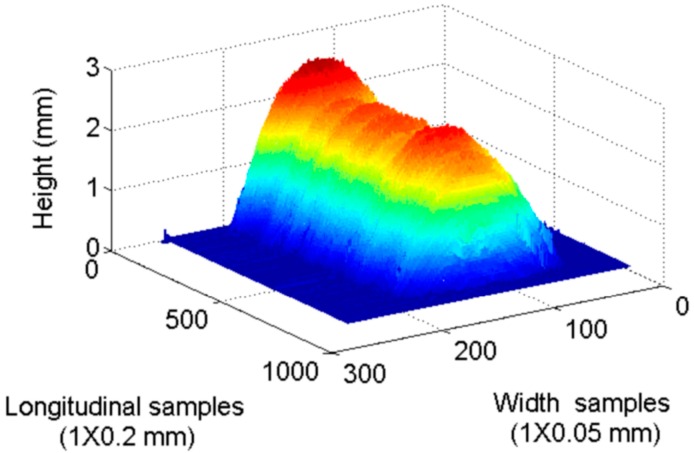
3D reconstruction of weld bead for a work point of 21 V and 6 m/min.

Figure 14 and Figure 15 present the frequency distribution of width and heigh for the previous example. A normal distribution can be clearly identified with more than 1000 data points per sample, so the normal distribution can be assumed in the rest of the work points used in validation process. Figure 16 shows a 3D reconstruction of the weld bead at the same work point. Table 1 and Table 2 show measurement results of width and height from the nine work points used in the validation, performed by the proposed vision system and by the 3D scanner, μ and *s* correspond, respectively, to the mean and standard deviation of the measurements in each work point.

In order to validate the reference point location and assuming a normal distribution in each measurement for all work points, Welch’s *t*-test was used to evaluate the null hypothesis, where the means of each corresponding measurement with the vision system and with the 3D scanner are equal. In this case, with different sample sizes for each measurement (1400 for the vision system and 700 for the 3D scanner), the Welch’s *t*-test was used in the unpaired form and for unequal variances. Table 3 shows the statistic |t| of Welch’s *t*-test computed for measurements of width and height (Table 1 and Table 2).

With more than 600 degrees of freedom and with interest only in the null hypothesis, the statistic is compared with a threshold chosen in the two-tailed Student’s t-distribution table. Table 3 shows that the values calculated for the Welch’s *t*-test do not exceed the threshold of α = 0.01, which is 2.5758 (using population size ∞ in the Student’s t-distribution table). So, the null hypothesis is not rejected and the means are equals, which validate the measurement methodology. In addition, Table 3 shows that, in general, larger values of Welch’s *t*-test are related to larger values of voltage. This is because a thresholding technique is used in the image processing methodology and the results of this process depends directly on the quantity of light present in the scene, which has greater proportionality with the arc size. So, in order to improve the measurement technique and without modifying the image processing methodology, the employment of other optical filters is suggested. In this case, it can be suggested to use a neutral density filter (no less than 80% transmission) and a band-pass optical filter in the region of infrared radiation (instead of the long-pass optical filter), aiming to reduce brightness and noise in the scene without affecting the location of actual reference points.

**Figure 17:**
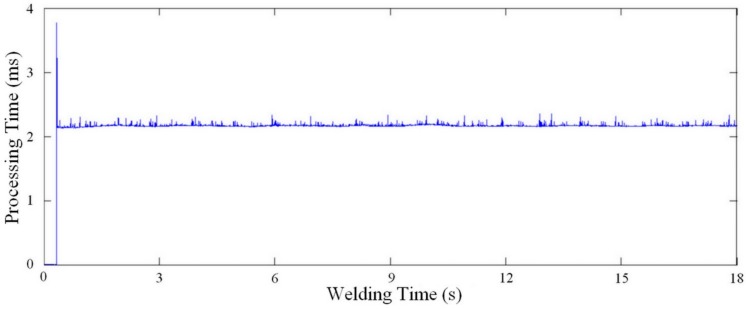
Processing time for each acquired image in the work point 21 V and 6 m/min [22].

Finally, Figure 17 plots the processing time for each image to obtain the width and height measurements with the work point of 21 V and 6 m/min. As shown in Figure 17, the processing time does not exceed 3 ms, this with a vision system built using C++ programming languages and a personal desktop computer with a 2.4 GHz processor and 3GB of RAM.

## 4. Conclusions

This paper proposes an approach for accomplishing real-time measurements of the weld bead width and height in GMAW processes by using a passive vision system and digital image processing techniques. From the results obtained from a comparison of the system using real-time data collected from different experimental work points with off-line 3D reconstruction of the weld beads, it is possible to conclude that the methodology proposed is efficient and it is ready to be used straightly in real-time control processes based on both the width and height of weld beads. Besides, the use of projective transformation mapping in the image calibration process allows the employment of a single camera without object occlusion, which is important to reduce processing time as well as to get a better setup in favor of industrial applications. Finally, the direct use of pixel positions and pixel binary values in the image processing methodology allows a shorter processing time, which is important in real-time applications that require additional processing, such as feedback control processes.

## Figures and Tables

**Table 1:** Measurement results of width.

Wire Feed Rate (m/min)	Voltage (V)	Width			
		Vision System		3D Scanner	
		µ (mm)	*s* (mm)	µ (mm)	*s* (mm)
3.9	21.0	6.16	0.38	6.18	0.37
4.5	23.0	6.87	0.36	6.92	0.48
4.5	19.0	5.69	0.27	5.72	0.28
6.0	23.8	7.80	0.35	7.85	0.46
6.0	21.0	6.79	0.34	6.83	0.38
6.0	18.2	5.84	0.43	5.87	0.60
7.5	23.0	8.16	0.50	8.22	0.53
7.5	19.0	6.23	0.77	6.30	0.78
8.1	21.0	7.70	0.45	7.71	0.29

**Table 2:** Measurement results of height.

Wire Feed Rate (m/min)	Voltage (V)	Height			
		Vision System		3D Scanner	
		µ (mm)	*s* (mm)	µ (mm)	*s* (mm)
3.9	21.0	1.59	0.17	1.58	0.14
4.5	23.0	1.67	0.14	1.66	0.10
4.5	19.0	1.69	0.11	1.70	0.09
6.0	23.8	1.97	0.15	1.95	0.17
6.0	21.0	2.15	0.09	2.16	0.12
6.0	18.2	2.31	0.16	2.29	0.18
7.5	23.0	2.39	0.17	2.37	0.17
7.5	19.0	2.87	0.20	2.85	0.22
8.1	21.0	2.48	0.18	2.50	0.17

**Table 3:** Welch’s *t*-test computed for measurements of width and height.

Wire Feed Rate (m/min)	Voltage (V)	Welch’s *t*-Test (|t|)	
		Width	Height
3.9	21.0	1.1361	1.4677
4.5	23.0	2.2514	2.0040
4.5	19.0	2.2676	2.2807
6.0	23.8	2.3471	2.5113
6.0	21.0	2.2441	1.8011
6.0	18.2	1.0838	2.3702
7.5	23.0	2.4013	2.4802
7.5	19.0	1.8946	1.9353
8.1	21.0	0.6760	2.4637
